# *Riemerella anatipestifer* infection in ducks induces IL-17A production, but not IL-23p19

**DOI:** 10.1038/s41598-019-49516-z

**Published:** 2019-09-13

**Authors:** Rochelle A. Flores, Cherry P. Fernandez-Colorado, Fahmida Afrin, Paula Leona T. Cammayo, Suk Kim, Woo H. Kim, Wongi Min

**Affiliations:** 1 College of Veterinary Medicine & Institute of Animal Medicine, Gyeongsang National University, Jinju, 52828 Korea; 2 Department of Veterinary Paraclinical Sciences, College of Veterinary Medicine, University of the Philippines Los Banos, College, Laguna, 4031 Philippines; 3 Animal Biosciences and Biotechnology Laboratory, Agricultural Research Service, United States Department of Agriculture, Beltsville, MD 20705 USA; 4 Present Address: Animal Genetic Resources Research Center, National Institute of Animal Science, Rural Development Administration, Hwang San-ro 1214-13, Unbong-up, Namwon 55717 Korea

**Keywords:** Cytokines, Cytokines, Bacterial infection, Bacterial infection

## Abstract

*R*. *anatipestifer* (RA) is one of the most harmful bacterial pathogens affecting the duck industry, and infection is associated with the production of proinflammatory cytokines, including IL-17A. Another proinflammatory cytokine, IL-23, is critical for the development of Th17 cells, which produce IL-17. However, IL-23 roles have not been studied in this infection. Here, we describe the identification and mRNA expression analysis of duck IL-23p19 (duIL-23p19) in splenic lymphocytes and macrophages stimulated with killed RA and in spleens of RA-infected ducks. Expression of duIL-23p19 transcript identified in this study was relatively high in livers of healthy ducks and was upregulated in mitogen-activated splenic lymphocytes as well as in splenic lymphocytes and macrophages stimulated with killed RA. In spleens of RA-infected ducks, expression levels of duIL-23p19 transcript were unchanged at all time points except on days 4 and 7 post-infection; however, duIL-17A and IL-17F expression levels were upregulated in both spleens of RA-infected ducks and splenic lymphocytes and macrophages stimulated with killed RA. In sera collected at 24 h after this infection, duIL-23p19 expression levels were unchanged, whereas IL-17A significantly upregulated. These results suggest that IL-23p19 does not play a critical role in the IL-17A response in early stages of RA*-*infected ducks.

## Introduction

*Riemerella anatipestifer* is a Gram-negative, non-motile, extracellular bacterium that belongs to the *Flavobacteriaceae* family, and infection of ducks with this pathogen causes acute and chronic septicaemia characterized by fibrinous polyserositis, and meningitis^1,2^. Currently, at least 21 *R*. *anatipestifer* strains that vary in virulence both between and sometimes within a given serotype have been identified and are characterized by a 5–75% mortality rate, depending on the virulence of the strain^2,3^. Although *R*. *anatipestifer* infection is a contagious disease that has resulted in significant economic losses in the duck industry^2^, little is known about the mechanisms of protective immune responses involved in *R*. *anatipestifer* pathogenesis.

Several attempts have been made to understand the host immune responses to *R*. *anatipestifer*. Using an immunoproteomic approach, immunoreactive proteins have been identified in duck or rabbit antisera to *R*. *anatipestifer*^4,5^. Upregulated host immunity was observed in ducks vaccinated with inactivated *R*. *anatipestifer* plus levamisole as an adjuvant^6^ or with recombinant *R*. *anatipestifer* outer membrane protein A plus CpG oligodeoxynucleotides as an adjuvant^7^. Furthermore, host genes involved in the immune response were identified in duck livers following *R*. *anatipestifer* infection^8^. Recently, comparative expression analyses of immune-related genes in ducks and chickens indicated that duck interleukin (IL)-17A was significantly increased in *R*. *anatipestifer*-infected ducks as well as in splenic lymphocytes activated with killed *R*. *anatipestifer*^9,10^. Decreased IL-17A expression and increased survival rate were observed in *R*. *anatipestifer*-infected berberine-treated ducks compared to infected untreated controls^11^.

The IL-17 family of cytokines consists of six structurally related members (IL-17A to IL-17F). IL-17A is the best characterized member of the IL-17 family and is produced mainly by IL-17A-producing CD4^+^ T cells, also called Th17 cells^12,13^, although this cytokine is now known to be produced by several other cell types, including macrophages, dendritic cells, natural killer T (NKT) cells, CD8^+^ T cells, γδ T cells, and neutrophils^14–16^. IL-17A is crucial for host protective immunity against various microbial pathogens. Specifically, the protective effects of IL-17 in host defence were demonstrated for *Escherichia coli*^17^, *Klebsiella pneumonia*e^18^, *Porphyromonas gingivalis*^19^, *Toxoplasma gondii*^20^, and *Candida albicans*^21^. Dysregulation of this cytokine is also known to contribute to development of tissue inflammation and autoimmune diseases, such as multiple sclerosis, rheumatoid arthritis, psoriasis, and inflammatory bowel disease^22–24^.

Th17 cell differentiation and maturation is influenced by the cytokine environment, including transforming growth factor beta (TGF-β), IL-1β, IL-6, IL-21, and IL-23^24,25^. IL-23 is an important factor in the inflammatory response to infection and is the upstream cytokine that primarily promotes Th17 differentiation and proliferation^26^. IL-23 is a heterodimeric cytokine composed of the cytokine subunits IL-23p19 (IL-23) and IL-12p40, which is also shared with IL-12. IL-23 can act in an IL-17-dependent and IL-17-independent manner^27,28^. In ducks, duck IL-17A shared 84% amino acid sequence identity with chicken IL-17A, approximately 46–47% to mammalian homologues. The genomic structure of duck IL-17A consisted of three exons and two introns was quite similar to its chicken and mammalian counterparts^29^. Until now, only partial information is available on duck IL-12p40 of which gene sequence (XM_021268516) predicts to encode a putative 317 amino acid protein. Our previous studies suggested that IL-17A is intimately associated with *R*. *anatipestifer* infection in ducks^9,11^. Thus, we were interested in elucidating any relationship between IL-23 and IL-17A during *R*. *anatipestifer* infection in ducks. Here, we provide the first description of a full-length duIL-23p19 cDNA and the expression profiles of duIL-23p19 transcript in various healthy tissues and mitogen-stimulated splenic lymphocytes using quantitative reverse transcription polymerase chain reaction (qRT-PCR). We also describe the comparative expression profiles of duIL-23p19 and related cytokines in duck splenic lymphocytes and macrophages stimulated with killed *R*. *anatipestifer* and in the spleens of *R*. *anatipestifer*-infected ducks.

## Results

### Molecular characterization of duIL-23p19

**Figure 1:**
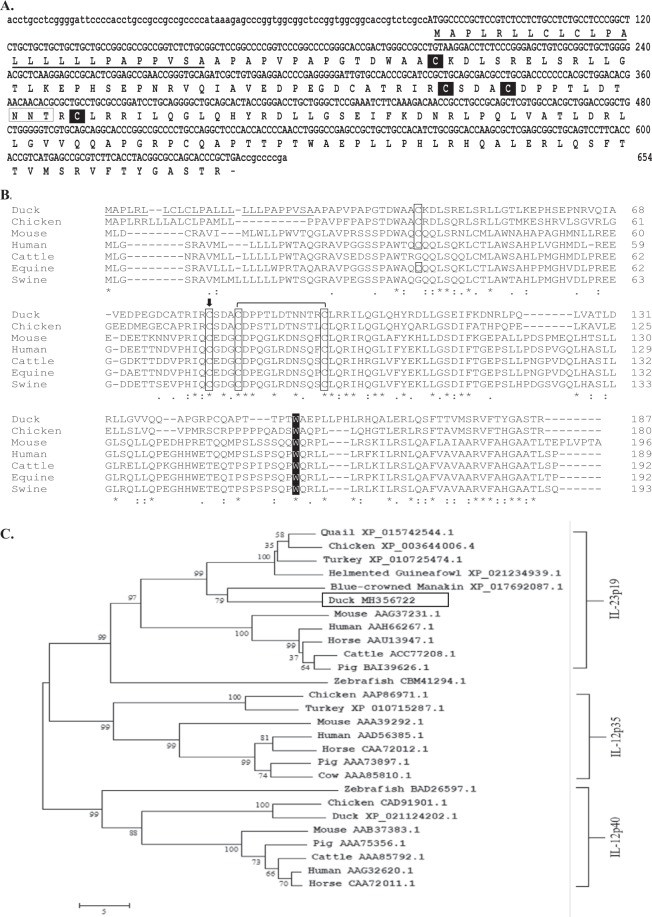
Molecular features and genetic analysis of duIL-23p19. (**A**) Nucleotide and deduced amino acid sequence of duIL-23p19. The predicted signal peptide region is underlined, the conserved cysteine residues are highlighted with black boxes, and the potential *N-*linked glycosylation site is boxed. (**B**) Multiple alignment of avian and mammalian IL-23p19-deduced amino acid sequences. Sequences were aligned using Clustal Omega software. Asterisks (*) indicate identical residues among sequences. Signal peptide of duIL-23p19 is underlined, and the conserved cysteine residues are boxed. The cysteine that interacts with the p40 subunit is indicated by an arrow head (↓), and the tryptophan (W) that binds with the signaling receptor is highlighted with black boxes. The GenBank accession numbers used in the comparison were duck (MH356722), chicken (XP_003644006.4), mouse (AAG37231.1), human (AAH66267.1), cattle (ACC77208.1), equine (AAU13947.1), and swine (BAI39626.1). (**C**) Phylogenetic tree indicates the relationship between the duck IL-23p19 amino acid sequences and those of other vertebrates. The tree was constructed with amino acid multiple alignments and the neighbour-joining method using the MEGA version 6 program. Node values represent percentage bootstrap confidence derived from 10,000 replicates. Accession number for each sequence is given after the species name. DuIL-23p19 is boxed.

The full-length cDNA encoding a duck homologue of chicken IL-23p19 was first cloned from ConA-stimulated splenic lymphocytes. The cloned duIL-23p19 sequence contained a 564-bp open reading frame (ORF) predicted to encode a putative 187 amino acid protein with a calculated molecular weight of 20.5 kDa (non-glycosylated) and an isoelectric point of 8.16. The duIL-23p19 amino acid sequence contains a signal sequence (amino acids 1–26), one potential N-linked glycosylation site (Asn-X-Ser/Thr) at position 94, and four cysteine residues involved in interchain disulphide bond formation (Fig. 1A,B). Analysis of the duIL-23p19 nucleotide sequence showed 37.8–66, 25.1–45.9, and 15.8% homology with that of poultry, mammals, and fish, respectively. Amino acid sequence comparison indicated that duIL-23p19 shares 40.4–62.1% amino acid sequence identity with avian counterparts, 31.5–36.1% with mammalian homologues, and 26.3% with the piscine equivalent (Table 1). Comparison of duIL-23p19 with IL-23p19 proteins of other species using the MEGA6 programs revealed that this protein is more closely related to avian species than to the corresponding mammalian and piscine proteins. Phylogenetic analysis indicated a distinct distance from IL-12p35 and IL-12p40 proteins (Fig. 1C).

**Table 1:** Duck IL-23p19 percent identity and similarity with IL-23p19 of other vertebrates.

Species	Duck IL-23p19				
	Nucleotide		Protein		
	Identity	GenBank Acc. No.	Identity	Similarity	GenBank Acc. No.
Human	42.9	BC066267.1	32.9	44.4	AAH66267.1
Mouse	25.1	AF301619.1	31.5	44.4	AAG37231.1
Horse	45.9	EU438773.1	36	46.9	ABZ91982.1
Cattle	32	NM_001205688.1	33	45.5	NP_001192617.1
Pig	41.6	NM_001130236.1	36.1	45.8	NP_001123708.1
Zebrafish	15.8	FN869917.1	26.3	38.5	CBM41294.1
Chicken	41.9	XM_003643958.4	60.1	71.5	XP_003644006.4
Guineafowl	37.8	XM_021379264.1	61.1	69.9	XP_021234939.1
Turkey	45.4	XM_010727172.2	40.4	49.5	XP_010725474.1
Common Starling	59.2	XM_014892528.1	52.8	62	XP_014748014.1
Ground Tit	65.7	XM_005532024.1	57.4	68.9	XP_005532081.1
Blue Crown Manakin	66.0	XM_017836598.1	62.1	72.1	XP_017692087.1
Golden Eagle	62.1	XM_011598153.1	54.8	63.1	XP_011596455.1

**Figure 2:**
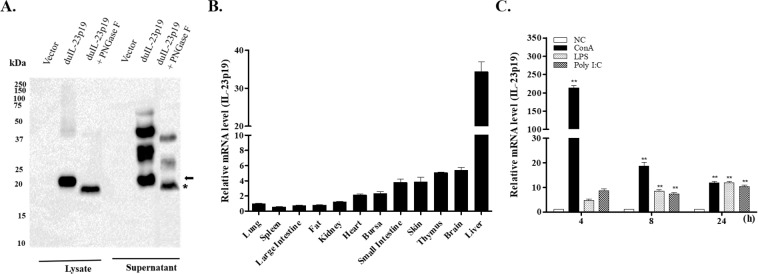
Molecular weight and expression of duIL-23p19 mRNA in healthy tissues and activated splenic lymphocytes. (**A**) Molecular weight detection of duIL-23p19 protein by Western blot analysis. COS-7 were transiently transfected with duIL-23p19-MYC construct or empty pcDNA3.1 vector. Cell lysates and supernatants were collected after 48 h and deglycosylated by 100 U peptide-*N*-glycosidase F (PNGase F) at 37 °C for 1 h. Lysates and supernatants were separated by SDS-PAGE under reducing conditions, and the specific bands were detected using anti-MYC antibody. The arrow and asterisk (*) indicate the specific bands and the specific deglycosylated band, respectively. (**B**) Distribution of duIL-23p19 in normal duck tissues. Total RNA was extracted from various tissues of 2-week-old healthy ducks. Tissue samples were pooled from five ducks and subjected to qRT-PCR analysis. Gene expression levels were normalized to those of β-actin and calibrated against the lowest expression level of the lung. Data are shown as the mean ± SE from two independent experiments. (**C**) DuIL-23p19 expression levels in mitogen-stimulated lymphocytes. Splenic lymphocytes were isolated from 2-week-old healthy ducks and activated with 10 μg/ml ConA, 10 μg/m LPS, or 25 μg/ml poly I:C for the indicated times. Gene expression levels were normalized to those of β-actin and calibrated with unstimulated cultured splenic lymphocytes (NC). Data are shown as the mean ± SE from two independent experiments performed in triplicate. **P < 0.01.

Following transfection of COS-7 cells with a construct expressing duIL-23p19-MYC, the molecular weight of duIL-23p19 was determined in PNGase F-treated lysates and supernatants. An 18.8-kDa protein (asterisk in Fig. 2A; calculated molecular weight of 20.5 kDa) is likely the backbone of duIL-23p19, and the protein of approximately 20.9 kDa (arrows) represents an *N*-linked glycosylated form in cell lysates and supernatants (Fig. 2A). The expression of the duIL-23p19 mRNA in various healthy tissues and mitogen-activated splenic lymphocytes was monitored by qRT-PCR. The duIL-23p19 mRNA was detected in all tested tissues. Compared to lung duIL-23p19 mRNA expression, the highest levels of this mRNA were detected in the liver, while moderate levels were found in the small intestine, skin, thymus, and brain. Moreover, very low levels of duIL-23p19 transcript were found in the fat, large intestine, and spleen (Fig. 2B). The expression level of duIL-23p19 transcript was generally upregulated in ConA-, LPS (*E*. *coli*)-, and poly I:C-stimulated splenic lymphocytes compared to unstimulated cultured splenic lymphocytes (Fig. 2C).

### mRNA expression profiles of IL-23 and Th17 cytokines in splenic lymphocytes and macrophages stimulated with killed *R*. *anatipestifer*

**Figure 3:**
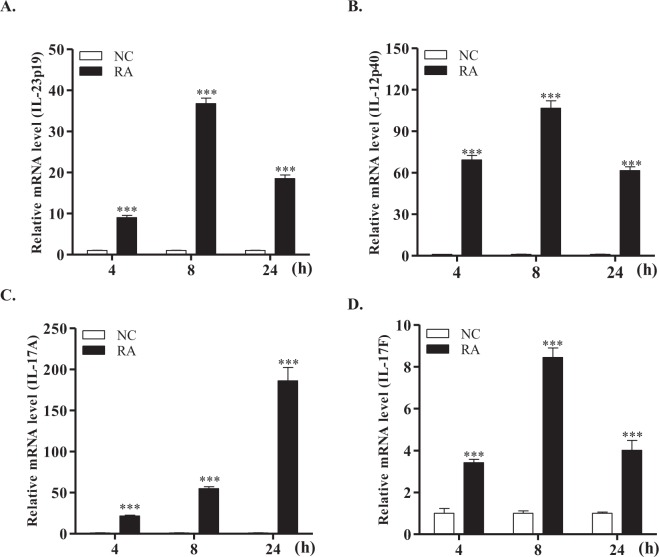
mRNA expression profiles of IL-23 and Th17 cytokines in splenic lymphocytes. Splenic lymphocytes were collected from 2-week-old healthy ducks and stimulated with killed *R*. *anatipestifer* serotype 7 for the indicated times. Samples were then subjected to qRT-PCR. The mRNA expression levels of IL-23p19 (**A**), IL-12p40 (**B**), IL-17A (**C**), and IL-17F (**D**) were normalized to those of β-actin and calibrated using the expression levels of untreated cultured lymphocytes (NC). Data are shown as the mean ± SE from three independent experiments performed in triplicate. ***P < 0.001. RA, *R*. *anatipestifer-*stimulated lymphocytes.

**Figure 4:**
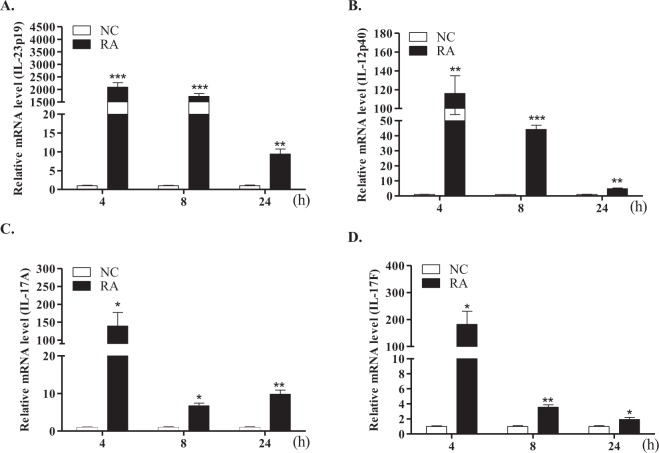
mRNA expression profiles of IL-23 and Th17 cytokines in duck macrophages. Macrophages were isolated from the spleens of 2-week-old healthy ducks and stimulated with killed *R*. *anatipestifer* serotype 7 for the indicated times. Samples were then subjected to qRT-PCR. mRNA expression levels of IL-23p19 (**A**), IL-12p40 (**B**), IL-17A (**C**), and IL-17F (**D**) were normalized to those of β-actin and calibrated using the expression levels of untreated cultured macrophages (NC). Data are shown as the mean ± SE from three independent experiments performed in triplicate. *P < 0.05, **P < 0.01, and ***P < 0.001. RA, *R*. *anatipestifer-*stimulated macrophages.

Our previous work indicated that IL-17A and IL-17F were significantly increased in ducks infected with *R*. *anatipestifer* and in duck splenic lymphocytes activated with killed *R*. *anatipestifer*^9,11^. IL-23p19 has been reported to play an important role in IL-17 production^27,28^; thus, the mRNA expression profiles of IL-23p19, IL-12p40, IL-17A, and IL-17F were investigated in splenic lymphocytes (Fig. 3) and macrophages (Fig. 4) stimulated with killed *R*. *anatipestifer*. All four cytokines were significantly higher at all time points in splenic lymphocytes and macrophages stimulated with killed *R*. *anatipestifer* compared with levels in unstimulated cultured controls. IL-23p19 expression showed 9–36.7-fold change in lymphocytes (Fig. 3A) and 9.4–2091.5-fold change in macrophages (Fig. 4A), while IL-12p40 expression showed a 61.5-106.6-fold change in lymphocytes (Fig. 3B) and a 4.8-116-fold change in macrophages (Fig. 4B). Furthermore, the expression levels of IL-17A and IL-17F transcripts were markedly upregulated in splenic lymphocytes (Fig. 3C,D) and macrophages (Fig. 4C,D) activated with killed *R*. *anatipestifer* compared to unstimulated cultured controls. These results suggested that both duIL-23p19 and IL-17A cytokines are significantly higher in splenic lymphocytes and macrophages treated with killed *R*. *anatipestifer*.

### mRNA expression profiles of IL-23 and Th17 cytokines in *R*. *anatipestifer*- infected ducks

**Figure 5:**
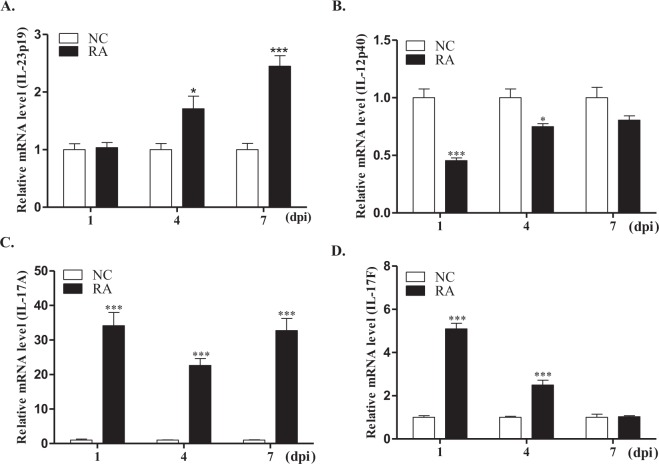
mRNA expression profiles of IL-23 and Th17 cytokines in the spleens of *R*. *anatipestifer*-infected ducks. Two-week-old healthy ducks were infected intramuscularly with 5 × 10^7^ CFU of *R*. *anatipestifer* serotype 7. Five ducks were sacrificed at each time point, and then the spleens were aseptically collected on 1, 4, and 7 days post-infection (dpi). The expression levels of IL-23p19 (**A**), IL-12p40 (**B**), IL-17A (**C**), and IL-17F (**D**) transcripts were quantified by qRT-PCR. Gene expression levels were normalized with β-actin and calibrated with expression levels from uninfected ducks (NC). The results from one representative experiment of two independent experiments are shown. Data are shown as the mean ± SE (n = 5). *P < 0.05, and ***P < 0.001. RA, splenocytes from *R*. *anatipestifer*-infected ducks.

The mRNA expression profiles of IL-23p19, IL-12p40, IL-17A, and IL-17F were investigated on days 1, 4, and 7 in the spleens of *R*. *anatipestifer*-infected ducks (Fig. 5). When compared to healthy controls, expression levels of duIL-23p19 mRNA were not different at day 1 after infection but were higher by 1.7-fold at day 4 and by 2.4-fold at day 7 post-infection (Fig. 5A). The mRNA expression levels of IL-12p40, the other subunit of IL-23, were lower at days 1 and 4, and unchanged at day 7 after infection in the spleens of *R*. *anatipestifer-*infected ducks (Fig. 5B). In addition, IL-17A and IL-17F expression levels were generally higher in *R*. *anatipestifer-*infected ducks (Fig. 5C,D).

### Expression of duIL-23p19 was not elevated at early stage of *R*. *anatipestifer* infection in ducks

**Figure 6:**
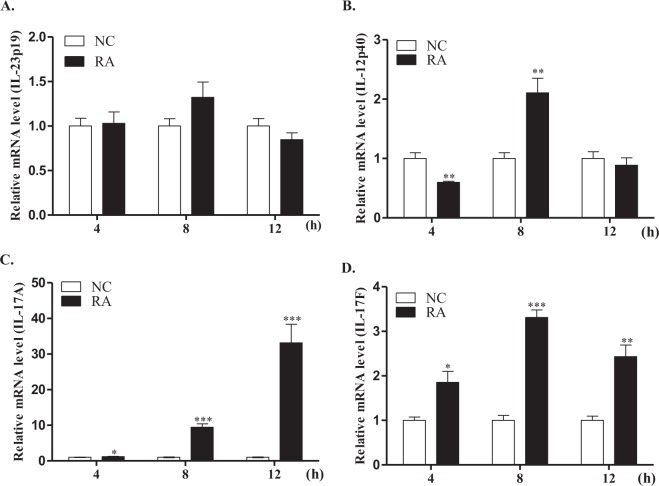
mRNA expression profiles of IL-23 and Th17 cytokines in the spleen at early time points in *R*. *anatipestifer*-infected ducks. Two-week-old healthy ducks were infected intramuscularly with 5 × 10^7^ CFU of *R*. *anatipestifer* serotype 7. Five ducks were sacrificed at each time point, and then the spleens were aseptically collected 4, 8, and 12 h post-infection. The expression levels of IL-23p19 (**A**), IL-12p40 (**B**), IL-17A (**C**), and IL-17F (**D**) transcripts were quantified by qRT-PCR. Gene expression levels were normalized with β-actin and calibrated with the expression levels in uninfected ducks (NC). The results from one representative experiment of two independent experiments are shown. Data are shown as the mean ± SE (n = 5). *P < 0.05, **P < 0.01, and ***P<0.001. RA, splenocytes from *R*. *anatipestifer*-infected ducks.

To examine the *in vivo* expression levels of duIL-23p19 during the early time points post-infection, the transcript expression profiles of IL-23p19, IL-12p40, IL-17A, and IL-17F in the spleen were investigated at 4, 8, and 12 h post-infection with *R*. *anatipestifer* (Fig. 6). When compared to healthy controls, IL-23p19 mRNA expression levels were unchanged at all time points in the spleen of *R*. *anatipestifer-*infected ducks (Fig. 6A). IL-12p40 mRNA expression levels showed a 0.6-2.1-fold change in the spleen of *R*. *anatipestifer-*infected ducks (Fig. 6B). However, IL-17A and IL-17F expression levels were higher at all time points post-infection in duck spleens (Fig. 6C,D).

**Figure 7:**
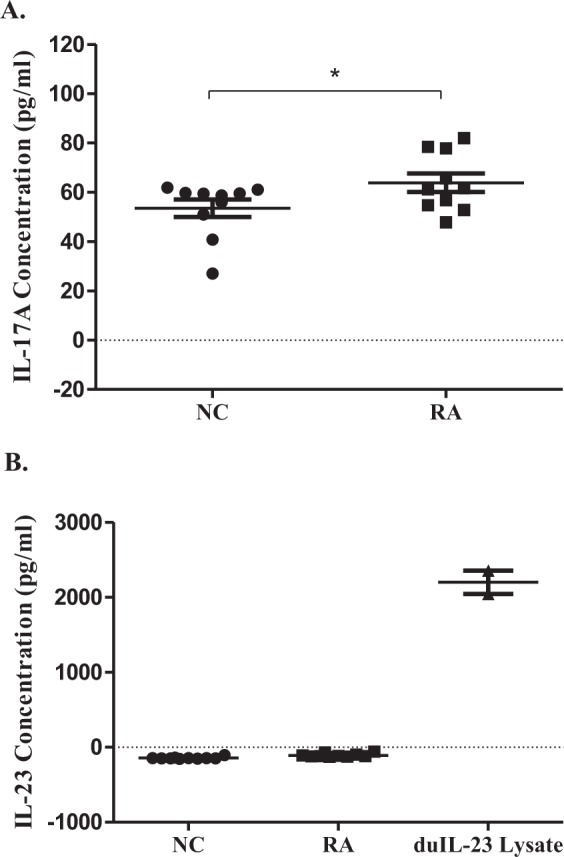
Serum IL-23p19 and IL-17A levels in *R*. *anatipestifer*-infected ducks. Two-week-old ducks were challenged intramuscularly with 5 × 10^7^ CFU of *R*. *anatipestifer* serotype 7 in 200 µl phosphate buffer saline (PBS). The uninfected control birds (NC) were administered 200 µl PBS intramuscularly. IL-17A and IL-23p19 levels in serum obtained at 24 h after infection were determined using the duck IL-17A and chicken IL-23 ELISA kits, respectively. COS-7 cells were transiently transfected with duIL-23p19-MYC construct. Cell lysates were collected after 48 h and used as positive control for chicken IL-23 ELISA. The results from one representative experiment of two independent experiments are shown. Data are shown as the mean ± SE (n = 10). *P < 0.05. RA, sera from *R*. *anatipestifer*-infected ducks.

Next, we investigated IL-17A and IL-23p19 expression in the sera of ducks at 24 h post-infection with *R*. *anatipestifer* (Fig. 7). When compared to healthy controls (53.5 ± 10.7), IL-17A expression levels were significantly higher in the sera of *R*. *anatipestifer-*infected ducks (63.8 ± 11.2) (Fig. 7A). However, IL-23p19 expression levels were unchanged post-infection in duck sera (Fig. 7B). Taken together, these observations suggested that expression levels of duIL-23p19 mRNA and protein, unlike IL-17A, are unchanged at early stage of *R*. *anatipestifer-*infected ducks.

## Discussion

IL-17A can be produced via IL-23-independent and -dependent mechanisms^27,30^. Our previous report suggested that IL-17A was significantly upregulated in ducks infected with *R*. *anatipestifer*^9,11^, a harmful bacterial pathogen that affects the duck farming industry. The IL-23/IL-17A pathogenic pathway has been implicated in organ-specific autoimmune inflammation^31^, psoriasis^32^, joint autoimmune inflammation^33^, colitis^34–36^, and hepatitis^37^. Therefore, this study was designed to investigate whether IL-23, which is the primary cytokine controlling Th17 development, is a critical immune factor in *R*. *anatipestifer* infection in ducks. To accomplish this, we first identified duIL-23p19 from ConA-stimulated duck splenic lymphocytes and described its molecular weight and mRNA expression profiles in healthy duck tissues as well as in *R*. *anatipestifer-*stimulated lymphoid cells and spleens of *R*. *anatipestifer*-infected ducks.

IL-23, which belongs to the IL-12 cytokine family, occurs as a heterodimer cytokine composed of a distinct p19 subunit (known as IL-23p19, IL-23 alpha subunit, or IL-23A) and a subunit to IL-12 (known as IL-12p40)^26^. In this study, we cloned the p19 subunit of IL-23 (duIL-23p19) and found that it contains a 564-bp ORF, predicted to encode 187 amino acids, including a 26-amino acid signal peptide and a single *N*-glycosylation site. The presence of a signal peptide in a sequence suggests that the protein is secreted via the classical pathway. The presence of a single predicted glycosylation site in the duck protein is in agreement with the presence of one putative *N*-glycosylation site in the chicken homologue^38^ and other avian species but is in contrast to the absence of an *N*-glycosylation site in the mammalian (human and mouse) counterparts^39^ and the five *N*-glycosylation sites in the zebrafish counterpart^40^. Interestingly, PNGase F-treated lysates and supernatants of COS-7 cells transfected with the duIL-23p19 construct displayed an 18.8-kDa protein, whereas the untreated cell lysates and supernatants exhibited an approximately 20.5-kDa protein (Fig. 2A), indicating the possibility of *N*-linked glycosylation in duIL-23p19.

Multiple sequence alignment of the deduced amino acid sequences of the IL-23p19 homologues from mammals, fish, and avians, including ducks, revealed conservation of amino acids in the cloned IL-23p19, particularly of the cysteine residues important for disulphide bond formation (C78/90 in human, C75/87 in zebrafish, and C86/98 in duck)^39^, the cysteines important for interacting with the IL-12p40 subunit (C82 in duck, C77 in swine, C74 in human, and C75 in mouse)^41^. In addition, the tryptophan residue is the signature hallmark of the binding site to the signaling receptors such as the gp130 cytokine family (W158 in human, W148 in zebrafish, and W153 in duck)^42^. Computer-assisted phylogenetic analysis showed that duIL-23p19 is more closely related to the identified IL-23p19 cytokines than IL-12p40 cytokines. Moreover, duIL-23p19 formed a branch in the same cluster with other avian IL-23p19 molecules. Amino acid comparison of duIL-23p19 also showed higher identities and similarities to avian species compared to its mammalian and piscine counterparts.

Two isoforms (p19a and p19b) of IL-23p19 cDNA were identified in both rainbow trout and Atlantic salmon. The expression levels of the two isoforms were low in most healthy uninfected tissues of rainbow trout and Atlantic salmon^43^. Expression levels of the p19a form were significantly upregulated in the spleen and head kidney of rainbow trout infected with the bacterial pathogen *Yersinia ruckeri* and in the head kidney of rainbow trout infected with viral haemorrhagic septicaemia virus (VHSV). In general, however, p19b expression was not significantly different between infected animals and time-matched uninfected controls^43^. In rainbow trout head kidney macrophages stimulated with poly I:C and peptidoglycan, p19a expression was high compared to p19b expression, which was not responsive or exhibited only a relatively small increase^43^. In our study, expression levels of the duIL-23p19 transcript were significantly upregulated in ConA-, LPS-, and poly I:C-stimulated splenic lymphocytes at all time points examined (Fig. 2C). Thus, our data indicate that duIL-23p19 cDNA more closely resembles the IL-23p19a form than the IL-23p19b form from rainbow trout. Moreover, the upregulated expression of duIL-23p19 in *R*. *anatipestifer-*stimulated splenic cells is in accord with the expression of IL-23p19 in *in vitro* LPS-stimulated zebrafish leucocytes^40^, chicken macrophage HD11 cells and CU91 T cells^38^, mouse peritoneal macrophages^44^, and human periodontal ligament cells^45^.

The proinflammatory cytokine IL-23 is essential for the differentiation and preservation of Th17 cells, which are the primary source of IL-17A^46^. Thus, to investigate the possibility of a connection between IL-23p19 and IL-17A, duck splenic lymphocytes and macrophages were treated with heat-killed *R*. *anatipestifer*. Expression levels of duIL-23p19 transcript, as well as IL-12p40, IL-17A, and IL-17F, were generally elevated in both immune cell types at all time points (Figs 3 and 4). Moreover, macrophages were more sensitive to this stimulation than lymphocytes. More specifically, duIL-123p19 expression at 4, 8 and 12 h following stimulation with *R*. *anatipestifer* was markedly upregulated by 9.0-, 36.7-, and 18.5-fold in the splenic lymphocytes and by 2091.5-, 1724.0-, and 9.4-fold in the splenic macrophages, respectively, indicating that macrophages are more sensitive than lymphocytes to *R*. *anatipestifer* stimulation. The primary sources of IL-23p19 and IL-17A are different. Lymphocytes, including CD4^+^αβ-T cells (Th17) and γδ-T cells, are primary sources of IL-17A, while IL-23p19 is produced by activated dendritic cells and macrophages^13,16,26,47^. In addition, human dendritic cells stimulated with killed gram-negative bacteria, but not with killed gram-positive bacteria, exhibited elevated expression of IL-23p19 transcripts^48^.

Unlike our *in vitro* results, expression levels of duIL-23p19 mRNA were unchanged at the early time points (4, 8, 12, and 24 h) of *R*. *anatipestifer* infection in ducks, although the expression levels were higher slightly by 1.7- and 2.4-fold at days 4 and 7 post-infection, whereas IL-17A mRNA expression levels were upregulated at all time points. Similar to mRNA expression patterns, expression levels of duIL-23p19 were unchanged in sera collected at 24 h after *R*. *anatipestifer* infection, whereas IL-17A expression levels were significantly upregulated (Fig. 7). Level of IL-23p19 in zebrafish infected with *Mycobacterium marinum* increased by 5-7-fold on day 1 and 4-fold on day 6 after infection^40^. After intraperitoneal injection of LPS in C57BL/6 J, plasma levels of IL-23p19 peaked as early as 3 h^44^. Expression levels of IL-23p19 in rainbow trout infected with *Yersinia ruckeri* were markedly upregulated 820-fold in spleens and 449-fold in head kidneys on day 1 after infection^43^. IL-23 has pathogenic roles in many autoimmune diseases^28^, while IL-23 is also important for protection against infections, such as *Listeria monocytogenes*^49^ and *Citrobacter rodentium*^50^.

In conclusion, we cloned the duIL-23p19 gene to determine whether the upregulation of IL-17A expression in *R*. *anatipestifer-*infected ducks is related to IL-23. Unlike splenic lymphocytes and macrophages stimulated with *R*. *anatipestifer*, expression levels of duIL-23p19 were not significantly upregulated at early time points in *R*. *anatipestifer*-infected ducks. These results suggest that during *R*. *anatipestifer* infection, IL-17A can have biological functions independent of duIL-23p19, especially at early times post-infection.

## Materials and Methods

### Animals and infection

Pekin ducklings (Joowon, ASTA Ducks, Korea) were housed in wire cages and raised in a temperature-controlled condition with constant light and had access to food and water *ad libitum* throughout the duration of the experiment. Sixty two-week-old ducks were challenged intramuscularly with 5 × 10^7^ CFU of *R*. *anatipestifer* serotype 7 in 200 µl phosphate buffer saline (PBS). The control birds were administered 200 µl PBS intramuscularly. *R*. *anatipestifer* used in this study were isolated from a commercial duck farm in Changwon, Gyeongnam Province, Korea, and serotyped at Chonbuk National University, Korea. The isolates were grown on agar plates supplemented with 5% sheep blood (Asan Pharmaceutical, Korea) for 48 h in a 37 °C incubator under 5% CO_2_. A single colony was inoculated into tryptic soy broth (TSB; Difco, USA) and incubated at 37 °C in a shaking incubator until logarithmic growth phase was achieved. The final concentration of the inoculum was determined by 10-fold serial dilutions on sheep blood agar plates. Spleens were collected from five ducks per group at 4, 8, 12, 24 (day 1), 96 (day 4), and 168 (day 7) h post-infection. All animal experiments were performed according to the guidelines of Gyeongsang National University (GNU) for the Care and Use of Experimental Animals and approved by the Institutional Animal Care and Use Committee (IACUC) of Gyeongsang National University (GNU-170725-C0031) (Jinju, Korea).

### Molecular cloning of duck IL-23p19

Primers (forward 5′ GGGGACAATGAAGGAGTCGC-3′ and reverse 5′ TCAGCGGGTGCTGGCGCC-3′) were designed from the predicted cDNA sequence of chicken IL-23p19 (Genbank accession number: XP_003644006.4) to obtain a partial sequence of duIL-23p19 cDNA. Based on the partial sequence information, 5′/3′ rapid amplification of cDNA ends (RACE) was performed to obtain the full-length duIL-23p19 cDNA using specific primers (Table 2), high-fidelity DNA polymerase (Bioneer, Korea), and the 5′/3′ RACE kit (2^nd^ Generation; Roche Applied Sciences, Germany) according to the manufacturer’s protocol. RT-PCR products were cloned to the TA Vector (RBC, Taiwan) and were sequenced (Macrogen, Korea). RT-PCR was performed on a DNA engine thermocycler (Bio-Rad, Hercules, CA, USA) with the following parameters: 5 min at 95 °C; 35 cycles of 1 min at 95 °C, 1 min at 55 °C, and 1 min at 72 °C; and a final 5-min extension at 72 °C.

**Table 2:** Primer sequences used for IL-23p19 cloning and quantitative RT-PCR of cytokine expression.

Target Genes	Purpose	Orientation and sequence (5′-3′)	References
IL-23p19	3′RACE	(For) TGGACACGAACAACACGCGC	In this study
	5′ RACE	(Rev) CGGCAGGCGGTTGTCTTTGA	
	RT-PCR	(For) ATGGCCCCGCTCCGTCTCCT (Rev) TCAGCGGGTGCTGGCGCCGT	In this study
	Real-Time RT-PCR	(For) AACCGGGTGCAGATCGCTGT (Rev) GCGCGTGTTGTTCGTGTCCA	In this study
IL-12p40	Real-Time RT-PCR	(For) CAGCTAATAGCCATGAAGTT (Rev) GTAGTTCTTTGCTTCACATT	52
IL-17A	Real-Time RT-PCR	(For) ATGTCTCCAACCCTTCGT (Rev) CCGTATCACCTTCCCGTA	54
IL-17F	Real-Time RT-PCR	(For) CTGAGAGACTTAATGGAGACTG (Rev) AGAATCTGAACGGCTGATG	54
β-actin	Real-Time RT-PCR	(For) GCTATGTCGCCCTGGATTTC (Rev) CACAGGACTCCATACCCAAGAA	55

### Sequence analysis

Protein sequence and amino acid alignment were analysed using Expert Protein Analysis System (ExPASy; www.expasy.org/tools/) and Clustal Omega multiple sequence alignment (www.ebi.ac.uk/Tools/msa/clustalo/), respectively. Percent identity and similarity of the duIL-23p19 sequence to its known and predicted vertebrate homologues were assessed using EMBOSS Needle pairwise sequence alignment (www.ebi.ac.uk/Tools/psa/emboss_needle). The signal peptide sequence was predicted using the SignalP 4.1 Server (www.cbs.dtu.dk/services/SignalP), and the potential *N-*linked glycosylation site was assessed using the NetNGlyc 1.0 Server (www.cbs.dtu.dk/services/NetNGlyc/). Theoretical pI value and predicted molecular weight were calculated using Compute pI/Mw (www.expasy.org/compute_pi). Evolutionary analysis was performed with a phylogenetic tree that was constructed using the neighbour-joining method in the MEGA6 program with a bootstrap value of 10,000^51^.

### Cell culture

Duck splenic lymphocytes were obtained from a two-week-old healthy duck as previously described^52^. Briefly, aseptically collected spleens of ducks were gently separated using a cell strainer (SPL Life Science, Korea) to obtain a single cell suspension, and the splenic lymphocytes were collected using Ficoll-Paque PLUS (GE Health Life Sciences, UK) following the manufacturer’s instructions. Cells were collected, washed twice with PBS, and then cultured in Dulbecco’s modified eagle’s medium (DMEM; Gibco Life Technologies, USA) supplemented with 10% foetal bovine serum (FBS) and 1% penicillin-streptomycin (10,000 unit/ml) in a 41 °C incubator under 5% CO_2_. Splenic lymphocytes were resuspended to a concentration of 5 × 10^6^ cells/ml and stimulated with 10 µg/ml concanavalin A (ConA; Amersham Bioscience, Sweden), 25 µg/ml polyinosinic:polycytidylic acid (poly I:C; Sigma-Aldrich, Germany), 10 µg/ml lipopolysaccharide (LPS from *Escherichia coli*, 0111:b4; Sigma-Aldrich), or heat-killed *R*. *anatipestifer* (1 × 10^6^ CFU/ml) for 4, 8, and 24 h. Duck primary macrophages were obtained from spleens of 2-week-old ducks as previously described^52,53^. Briefly, spleens were homogenized and sieved using a syringe plunger and passed through a cell strainer to obtain a single cell suspension. Cells were collected, washed twice with PBS and resuspended in a culture flask with DMEM supplemented with 10% FBS and penicillin-streptomycin (10,000 unit/ml). Cells were then incubated in a 41 °C incubator under 5% CO_2_ for 2 days to allow adherence of cells. Non-adherent cells were removed by washing with DMEM, and adherent cells were cultured until reaching 80% confluency. Adherent cells were seeded (5 × 10^6^ cells/ well) and were treated with heat-killed *R*. *anatipestifer* (1 × 10^6^ CFU/ml) for 4, 8, and 24 h. Heat killed *R*. *anatipestifer* was prepared by placing in a water bath at 100 °C for 5 min^9,11^. COS-7 cells were grown as described above in a 37 °C incubator under 5% CO_2_.

### Plasmid construction and cell transfection

The sequence of duIL-23p19 cDNA with a MYC-tag for expression (duIL-23p19-MYC) was amplified by PCR from single-stranded cDNA using the following specific primers: forward 5′-GATCAAGCTTATGGCCCCGCTCCGTCTCCT-3′ and reverse 5′-GATCGAATTCTCACAGATCCTCTTCTGAGATGAGTTTTTGTTCGCGGGTGCTGGCGCCGTAGG-3′. The primers included *Hind*III and *Eco*RI restriction enzyme sites, indicated by a single solid underline, and a MYC-expressing sequence, indicated by a dashed underline. PCR products were purified using the FavorPrep GEL/PCR Purification Mini Kit (Favorgen, Taiwan), digested with *Hind*III and *Eco*RI, ligated into the matching restriction enzyme sites of the pcDNA 3.1 + vector (Invitrogen, Waltham, MA, USA), transformed into DH5α competent cells (RBC), and sequenced for confirmation (Macrogen). COS-7 cells were transiently transfected with 10 µg of duIL-23p19-MYC construct or empty vector as a negative control using FuGene 6 transfection reagent (Promega, Madison, WI, USA) following the manufacturer’s instructions. Transfected cells were grown in Opti-MEM^®^ I (Gibco Life Technologies, USA), a serum-free medium, for 48 h at 37 °C in 5% CO_2_.

### Western blot analysis

Cell lysates and supernatants of the COS-7 cells transfected with duIL-23p19-MYC construct or empty vector were collected after incubation. First, cells were lysed with ice-cold lysis buffer with 1% Halt protease inhibitor cocktail (Thermo Fisher Scientific, Waltham, MA, USA) and incubated overnight at 4 °C. Cell lysates and supernatants were centrifuged at 12,000 rpm for 30 min at 4 °C to remove cell debris. Samples were mixed with an equal volume of sample buffer (0.125 M Tris-HCl pH 6.8; 4% SDS; 20% glycerol; 10% 2-mercaptoethanol; 0.004% bromophenol blue), treated with peptide *N-*glycosidase F (PNGase F; New England Biolabs, Ipswich, MA, USA) when necessary, and then heated at 95 °C for 5 min. Proteins were separated on sodium dodecyl sulfate polyacrylamide gel electrophoresis (SDS-PAGE) gels and then transferred to polyvinylidene difluoride (PVDF; Bio-Rad) membranes. The membranes were blocked with 4% nonfat dry milk (Sigma-Aldrich, St. Louis, MO, USA) in PBS containing 0.05% Tween-20 (PBS-T) for 2 h at room temperature and then incubated with monoclonal anti-MYC mouse antibody (Cell Signaling Technology, Danvers, MA, USA) overnight at 4 °C. The membranes were then washed five times with PBS-T and incubated with horseradish peroxidase-conjugated goat anti-mouse IgG antibody (Promega) in PBS-T with 4% nonfat dry milk for 1 h at room temperature. The membranes were washed with PBS-T five times, incubated with chemiluminescent reagent EZ West Lumi plus (Dogen Bio Co., LTD, Korea), and visualized using the ChemiDoc Imaging System (Bio-Rad).

### qRT-PCR

Spleens, splenic lymphocytes, and macrophages from ducks infected with *R*. *anatipestifer* and time-matched unstimulated and uninfected control samples were subjected to qRT-PCR in duplicate. Samples were collected and homogenized using a grinder (Dalhan Sci., Korea) for spleens and a vortex for cells. Total RNA was isolated from the samples using RiboEx (GeneAll, Korea), purified with RNeasy mini kit (Qiagen), and stored at −70 °C. The total RNA concentration was measured using a nano-spectrophotometer (Optizen, Korea) and treated with DNase I to remove contaminating genomic DNA. A single-strand cDNA was synthesized from the total RNA using random hexamer primers and the Quantitect reverse transcription kit (Qiagen). Then, qRT-PCR analysis was performed in duplicate using CFX 96 real-time PCR system (Bio-Rad) with SYBR Green (Bioneer) and gene-specific primers (Table 2). In order to verify the presence of a single amplification product without primer dimers, a melting curve was obtained at the end of each amplification program. Relative expression levels of individual transcripts from infected ducks and stimulated splenic lymphocytes and macrophages were compared with those of the control uninfected ducks and unstimulated lymphoid cells using Bio-Rad CFX software respectively and were quantified using the 2^−ΔΔ^CT method with β-actin as a reference gene for normalization.

### Enzyme linked immunosorbent assay (ELISA)

Two-week-old ducks were challenged intramuscularly with 5 × 10^7^ CFU of *R*. *anatipestifer* serotype 7 in 200 µl phosphate buffer saline (PBS). The control birds were administered 200 µl PBS intramuscularly. Whole blood was obtained after 24 h, and serum was separated after clotting by centrifugation. Aliquots were then taken and stored at −70 °C until test were performed. Serum IL-17A and IL-23 levels were determined by duck IL-17A and chicken IL-23 ELISA kits (MyBioSource, USA), respectively, according to manufacturer’s instructions.

### Statistical analysis

Data were analysed using the Student’s *t*-test or one-way analysis of variance (ANOVA) followed by Dunnett’s multiple comparison test using InStat® software (GraphPad, USA). Differences were considered statistically significant when *P < 0.05, **P < 0.01, or ***P < 0.001. The data were expressed as the mean values ± standard error (SE).

## Data Availability

The datasets generated during and/or analysed during the current study are available with the corresponding author, and can be accessed on reasonable request.
